# 1,3,6-Trihy­droxy-7-meth­oxy-2,8-bis­(3-methyl­but-2-en­yl)-9*H*-xanthen-9-one

**DOI:** 10.1107/S1600536810049123

**Published:** 2010-11-30

**Authors:** Gwendoline Cheng Lian Ee, Wei Chung Sim, Huey Chong Kwong, Mohamed Ibrahim Mohamed Tahir, Sidik Silong

**Affiliations:** a Department of Chemistry, Faculty of Science, Universiti Putra Malaysia, 43400 UPM Serdang, Selangor, Malaysia

## Abstract

The title compound (trivial name α-mangostin), C_24_H_26_O_6_, isolated from *Cratoxylum glaucum*, is characterized by a xanthone skeleton of three fused six-membered rings and two 3-methyl­but-2-enyl side chains. The three rings in the structure are nearly coplanar, with an r.m.s. deviation for the tricyclic ring system of 0.0014 Å. The two 3-methyl­but-2-enyl side chains are in (+)-synclinal and (-)-anti­clinal conformations. Intra­molecular O—H⋯O and C—H⋯O inter­actions occur. The crystal structure is stabilized by inter­molecular O—H⋯O, C—H⋯O and C—H⋯π inter­actions.

## Related literature

For standard bond lengths, see Allen *et al.* (1987 ▶). For related structures, see: Marek *et al.* (2003 ▶); Ndjakou *et al.* (2007 ▶); Boonnak *et al.* (2007 ▶). For the biological activity of *Cratoxylum* species, see: Boonnak *et al.* (2006 ▶); Bennett *et al.* (1993 ▶); Nguyen & Harrison (1998 ▶); Boonsri *et al.* (2006 ▶); Reutrakul *et al.* (2006 ▶). 
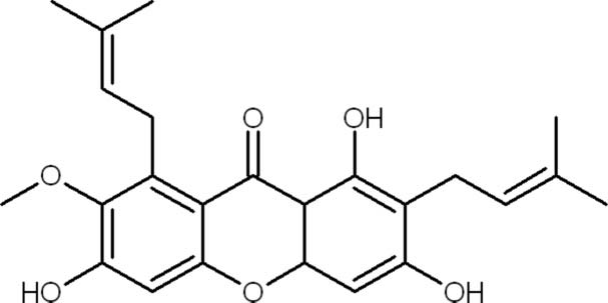


## Experimental

### 

#### Crystal data

C_24_H_26_O_6_
*M* *_r_* = 410.47
Orthorhombic, 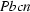

*a* = 14.6818 (3) Å
*b* = 9.53505 (19) Å
*c* = 29.8893 (6) Å
*V* = 4184.24 (14) Å^3^
*Z* = 8
Cu *K*α radiation
μ = 0.77 mm^−1^
*T* = 150 K
0.11 × 0.10 × 0.04 mm

#### Data collection

Oxford Diffraction Gemini E diffractometer
Absorption correction: multi-scan (*CrysAlis PRO*; Oxford Diffraction, 2006 ▶) *T*_min_ = 0.926, *T*_max_ = 0.970
13773 measured reflections
4032 independent reflections
2812 reflections with *I* > 2σ(*I*)
*R*_int_ = 0.034

#### Refinement

*R*[*F* ^2^ > 2σ(*F* ^2^)] = 0.041
*wR*(*F* ^2^) = 0.113
*S* = 0.88
4018 reflections
271 parameters
H-atom parameters constrained
Δρ_max_ = 0.34 e Å^−3^
Δρ_min_ = −0.27 e Å^−3^

### 

Data collection: *CrysAlis CCD* (Oxford Diffraction, 2006 ▶); cell refinement: *CrysAlis CCD*; data reduction: *CrysAlis RED* (Oxford Diffraction, 2006 ▶); program(s) used to solve structure: *SIR92* (Altomare *et al.*, 1994 ▶); program(s) used to refine structure: *CRYSTALS* (Betteridge *et al.*, 2003 ▶); molecular graphics: *Mercury* (Macrae *et al.*, 2006 ▶); software used to prepare material for publication: *CRYSTALS*.

## Supplementary Material

Crystal structure: contains datablocks global, I. DOI: 10.1107/S1600536810049123/kp2293sup1.cif

Structure factors: contains datablocks I. DOI: 10.1107/S1600536810049123/kp2293Isup2.hkl

Additional supplementary materials:  crystallographic information; 3D view; checkCIF report

## Figures and Tables

**Table 1:** Hydrogen-bond geometry (Å, °) *CgA* is the mid-point of the C14=C15 double bond.

*D*—H⋯*A*	*D*—H	H⋯*A*	*D*⋯*A*	*D*—H⋯*A*
C13—H132⋯O5	0.94	2.24	2.885 (3)	125
C14—H141⋯O10^i^	0.96	2.36	3.304 (3)	168
O21—H211⋯O5	0.85	1.71	2.499 (3)	155
O10—H101⋯O21^ii^	0.84	1.88	2.691 (3)	164
O30—H301⋯*CgA*	0.86	2.38	3.227 (3)	166

Symmetry codes: (i) 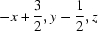; (ii) 
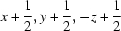
.

**Fig. 1.**
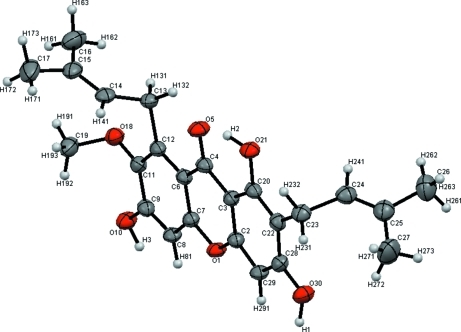
Molecular structure of I with atom numbering and displacement ellipsoids at 50% probablity level.

**Fig. 2.**
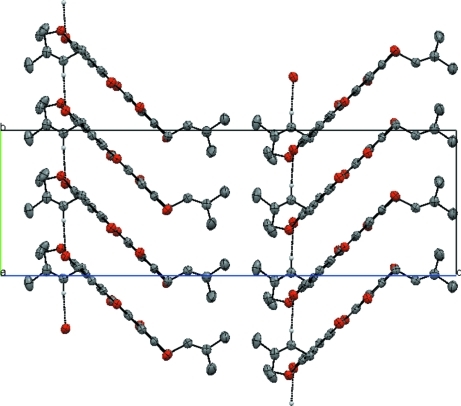
View of the chain of hydrogen bonding along a axis. Symmetry codes used (-x + 3/2, y - 1/2, z).

**Fig. 3.**
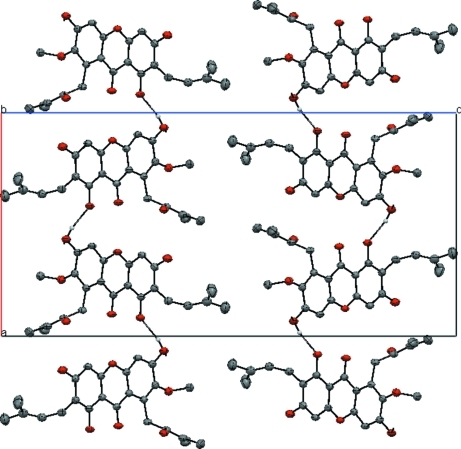
View of the chain of hydrogen bonding along b axis. Symmetry codes used (x + 1/2, y + 1/2, -z + 1/2).

## supplementary crystallographic information

### Comment

*Cratoxylum* is a small genus which comprises of six species indigenous to Southeast Asia (Boonnak *et al.*, 2006). The stem bark of the species has been applied in traditional medicine in Malaysia (Bennett *et al.*, 1993). Reports indicated that the bark, roots and leaves have been used in folk medicine to treat fevers, cough, diarrhoea, itches, ulcers and abdominal complaints (Nguyen & Harrison, 1998). Antibacterial, cytotoxic and anti-HIV constituents have also been reported in recent studies on *Cratoxylum* species (Boonsri *et al.*, 2006; Reutrakul *et al.*, 2006). In this report, the X-ray crystallographic structure for the title compound 1,3,6-trihydroxy-7-methoxy-2,8-bis(3-methylbut-2-enyl)-4aH-xanthen-9(9aH)-one, α-mangostin (I) isolated from *Cratoxylum glaucum*  is reported.

Bond distances and angles in the title compound (Fig. 1) are in a normal range (Allen *et al.*, 1987) and are comparable with those for closely related structures (Marek *et al.*, 2003; Ndjakou *et al.*, 2007; Boonnak *et al.*, 2007). The tricyclic ring system (rings A, B and C) is nearly planar (r. m. s. deviation of 0.0014 Å). Atoms O10, O18, O21 and O30 deviate from this plane by 0.045, 0.056, 0.025 and 0.071 Å, respectively. The dihedral angle between the rings A and B is 1.51°, and between the rings B and C is 1.73°.

The orientations of two 3-methylbut-2-enyl side chains are defined by their respective torsion angles: the first side chain towards the ring A with the atom sequence C11—C12—C13—C14 of 80.19° [116.4° in similar compound (Ndjakou *et al.*, 2007)] indicating a (+)-synclinal conformation; the second side chain towards the ring C with the atom sequence C20—C22—C23—C24 of -87.62°, indicating a (-)-anticlinal conformation. The average value of C—O1 bond lengths in pyranoid ring B is 1.368 Å. The crystal structure is stabilised by intra- and intermolecular O—H···O and C—H···O hydrogen bonding (Table 1, Figs. 2 and 3). In addition to classical hydrogen bonds, there is a contact from hydroxyl group O30—H301 to centroid (Cg A) of the C14═C15 (H301···Cg A= 2.384 Å, O30···Cg A=3.227 Å and O30—H301···Cg A = 166.2.

### Experimental

The stem bark (3.75 kg) of *Cratoxylum glaucum* was ground and extracted with *n*-hexane, ethyl acetate and methanol. Fractionation of the ethyl acetate extract with vacuum liquid chromatography over Merck 7731 silica gel produced 25 fractions. Fraction 8 was subjected to further purification by column chromatography (Merck Kieselgel No. 1.09385.1000). These columns were eluted with hexane, hexane/ethyl acetate, ethyl acetate/methanol in a step-wise gradual increment in polarity. The yellow amorphous powder obtained from fraction 6 was dissolved in chloroform and left for a month before orange crystals were obtained. The melting point was 452-453 K.

### Refinement

The H atoms were all observed in a difference map, but those attached to carbon atoms were positioned geometrically. The H atoms were initially refined with soft restraints on the bond lengths and angles to regularize their geometry (C—H in the range 0.93–0.98, N—H in the range 0.86–0.89 N—H to 0.86 O—H = 0.82 Å) and *U*_iso_(H) (in the range 1.2–1.5 times *U*_eq_ of the parent atom), after which the positions were refined with riding constraints.

### Crystal data

**Table:**

C_24_H_26_O_6_	*D*_x_ = 1.303 Mg m^−^^3^
*M**_r_* = 410.47	Melting point: 452 K
Orthorhombic, *P**b**c**n*	Cu *K*α radiation, λ = 1.54184 Å
Hall symbol: -P 2n 2ab	Cell parameters from 5947 reflections
*a* = 14.6818 (3) Å	θ = 3.0–71.2°
*b* = 9.53505 (19) Å	µ = 0.77 mm^−^^1^
*c* = 29.8893 (6) Å	*T* = 150 K
*V* = 4184.24 (14) Å^3^	Plate, yellow
*Z* = 8	0.11 × 0.10 × 0.04 mm
*F*(000) = 1744	

### Data collection

**Table:**

Oxford Diffraction Gemini E diffractometer	4032 independent reflections
Radiation source: sealed x-ray tube	2812 reflections with *I* > 2σ(*I*)
graphite	*R*_int_ = 0.034
ω/2θ scans	θ_max_ = 71.4°, θ_min_ = 4.2°
Absorption correction: multi-scan (*CrysAlis PRO*; Oxford Diffraction, 2006)	*h* = −17→17
*T*_min_ = 0.926, *T*_max_ = 0.970	*k* = −11→11
13773 measured reflections	*l* = −36→29

### Refinement

**Table:**

Refinement on *F*^2^	Primary atom site location: structure-invariant direct methods
Least-squares matrix: full	Hydrogen site location: inferred from neighbouring sites
*R*[*F*^2^ > 2σ(*F*^2^)] = 0.041	H-atom parameters constrained
*wR*(*F*^2^) = 0.113	Method = Modified Sheldrick *w* = 1/[σ^2^(*F*^2^) + (0.08*P*)^2^ + 0.0*P*], where *P* = [max(*F*_o_^2^,0) + 2*F*_c_^2^]/3
*S* = 0.88	(Δ/σ)_max_ = 0.0003
4018 reflections	Δρ_max_ = 0.34 e Å^−^^3^
271 parameters	Δρ_min_ = −0.27 e Å^−^^3^
0 restraints	

### Special details

**Table:**

Refinement. (sinθ X)2 was set to > 0.01 to eliminate reflection measured near the vicinity of the beam stop.

### Fractional atomic coordinates and isotropic or equivalent isotropic displacement parameters (Å^2^)

**Table:**

	*x*	*y*	*z*	*U*_iso_*/*U*_eq_	
O1	0.87635 (7)	0.30339 (12)	0.25523 (4)	0.0309	
C2	0.82099 (10)	0.22222 (17)	0.28118 (5)	0.0276	
C3	0.72644 (10)	0.23373 (17)	0.27827 (5)	0.0279	
C4	0.68351 (10)	0.32642 (17)	0.24630 (5)	0.0285	
O5	0.59844 (7)	0.32795 (13)	0.24258 (4)	0.0368	
C6	0.74439 (10)	0.41346 (17)	0.21923 (5)	0.0274	
C7	0.83874 (10)	0.39435 (17)	0.22495 (5)	0.0274	
C8	0.90312 (10)	0.46582 (18)	0.19999 (6)	0.0307	
C9	0.87454 (10)	0.55982 (17)	0.16816 (6)	0.0307	
O10	0.93348 (7)	0.63408 (13)	0.14256 (4)	0.0361	
C11	0.78053 (10)	0.58382 (17)	0.16158 (5)	0.0292	
C12	0.71573 (10)	0.51369 (18)	0.18686 (5)	0.0286	
C13	0.61617 (10)	0.55256 (18)	0.17851 (6)	0.0305	
C14	0.58048 (10)	0.47960 (19)	0.13754 (6)	0.0329	
C15	0.54434 (11)	0.5383 (2)	0.10128 (6)	0.0416	
C16	0.52901 (15)	0.6929 (3)	0.09490 (8)	0.0574	
C17	0.51697 (14)	0.4474 (3)	0.06219 (7)	0.0634	
O18	0.75300 (8)	0.68622 (13)	0.13202 (4)	0.0356	
C19	0.76973 (12)	0.6541 (2)	0.08604 (6)	0.0444	
C20	0.67452 (10)	0.14669 (17)	0.30706 (5)	0.0291	
O21	0.58222 (7)	0.15647 (13)	0.30532 (4)	0.0349	
C22	0.71415 (10)	0.05261 (18)	0.33651 (5)	0.0302	
C23	0.65776 (11)	−0.03859 (19)	0.36731 (6)	0.0337	
C24	0.63628 (11)	0.0336 (2)	0.41077 (6)	0.0370	
C25	0.65495 (12)	−0.0099 (2)	0.45188 (7)	0.0441	
C26	0.62764 (17)	0.0757 (3)	0.49190 (8)	0.0589	
C27	0.7041 (2)	−0.1435 (3)	0.46259 (9)	0.0688	
C28	0.81001 (11)	0.04585 (18)	0.33691 (6)	0.0309	
C29	0.86367 (10)	0.12942 (18)	0.30953 (6)	0.0311	
O30	0.84808 (8)	−0.04872 (13)	0.36537 (4)	0.0388	
H81	0.9656	0.4502	0.2049	0.0363*	
H131	0.6130	0.6516	0.1755	0.0361*	
H132	0.5811	0.5267	0.2037	0.0360*	
H141	0.5857	0.3795	0.1381	0.0387*	
H161	0.5550	0.7236	0.0664	0.0850*	
H163	0.5558	0.7469	0.1192	0.0852*	
H162	0.4643	0.7120	0.0934	0.0857*	
H171	0.4551	0.4673	0.0536	0.0945*	
H173	0.5552	0.4712	0.0368	0.0938*	
H172	0.5250	0.3454	0.0687	0.0952*	
H191	0.7492	0.7303	0.0678	0.0666*	
H193	0.8329	0.6388	0.0802	0.0658*	
H192	0.7357	0.5710	0.0777	0.0666*	
H231	0.6891	−0.1275	0.3725	0.0390*	
H232	0.6016	−0.0609	0.3524	0.0393*	
H241	0.6065	0.1214	0.4085	0.0447*	
H261	0.6799	0.1001	0.5095	0.0886*	
H263	0.5951	0.1616	0.4833	0.0880*	
H262	0.5893	0.0198	0.5113	0.0878*	
H271	0.7562	−0.1207	0.4801	0.1025*	
H273	0.7216	−0.1943	0.4357	0.1020*	
H272	0.6645	−0.2019	0.4808	0.1027*	
H291	0.9267	0.1242	0.3103	0.0351*	
H211	0.5707	0.2133	0.2842	0.0526*	
H101	0.9842	0.6299	0.1553	0.0544*	
H301	0.9064	−0.0397	0.3648	0.0578*	

### Atomic displacement parameters (Å^2^)

**Table:**

	*U*^11^	*U*^22^	*U*^33^	*U*^12^	*U*^13^	*U*^23^
O1	0.0172 (5)	0.0397 (6)	0.0357 (6)	0.0002 (4)	0.0001 (4)	0.0033 (5)
C2	0.0216 (7)	0.0330 (9)	0.0283 (8)	−0.0023 (6)	0.0008 (6)	−0.0033 (7)
C3	0.0220 (7)	0.0323 (8)	0.0295 (8)	−0.0012 (6)	−0.0005 (6)	−0.0042 (7)
C4	0.0207 (7)	0.0344 (8)	0.0303 (8)	−0.0001 (6)	0.0005 (6)	−0.0060 (7)
O5	0.0177 (5)	0.0478 (7)	0.0447 (7)	−0.0010 (5)	−0.0011 (5)	0.0055 (6)
C6	0.0213 (7)	0.0333 (8)	0.0277 (8)	−0.0005 (6)	0.0000 (6)	−0.0045 (6)
C7	0.0215 (7)	0.0320 (8)	0.0288 (8)	0.0007 (6)	−0.0009 (6)	−0.0023 (7)
C8	0.0171 (6)	0.0392 (9)	0.0357 (9)	−0.0008 (6)	−0.0007 (6)	−0.0030 (7)
C9	0.0242 (7)	0.0370 (9)	0.0309 (8)	−0.0037 (6)	0.0018 (6)	−0.0036 (7)
O10	0.0231 (5)	0.0468 (7)	0.0385 (7)	−0.0043 (5)	−0.0014 (5)	0.0069 (5)
C11	0.0255 (7)	0.0330 (8)	0.0290 (8)	0.0002 (6)	−0.0016 (6)	−0.0030 (7)
C12	0.0215 (7)	0.0351 (9)	0.0292 (8)	0.0012 (6)	−0.0009 (6)	−0.0053 (7)
C13	0.0209 (7)	0.0373 (9)	0.0335 (8)	0.0041 (6)	−0.0001 (6)	0.0011 (7)
C14	0.0191 (7)	0.0415 (9)	0.0381 (9)	0.0006 (6)	0.0003 (6)	−0.0012 (8)
C15	0.0220 (7)	0.0669 (13)	0.0358 (9)	0.0017 (7)	−0.0005 (7)	0.0024 (9)
C16	0.0424 (10)	0.0754 (15)	0.0545 (13)	0.0110 (10)	−0.0030 (9)	0.0238 (12)
C17	0.0384 (10)	0.111 (2)	0.0411 (12)	−0.0001 (11)	−0.0084 (9)	−0.0129 (13)
O18	0.0296 (5)	0.0420 (6)	0.0351 (6)	0.0015 (5)	−0.0008 (5)	0.0037 (5)
C19	0.0322 (9)	0.0691 (13)	0.0319 (9)	−0.0004 (8)	−0.0003 (7)	0.0081 (9)
C20	0.0205 (7)	0.0361 (8)	0.0309 (8)	−0.0021 (6)	0.0009 (6)	−0.0076 (7)
O21	0.0185 (5)	0.0470 (7)	0.0391 (7)	−0.0024 (4)	0.0005 (4)	0.0046 (5)
C22	0.0272 (7)	0.0342 (9)	0.0292 (8)	−0.0025 (6)	0.0003 (6)	−0.0042 (7)
C23	0.0267 (7)	0.0386 (9)	0.0357 (9)	−0.0044 (6)	−0.0003 (6)	0.0009 (8)
C24	0.0295 (8)	0.0401 (10)	0.0413 (10)	−0.0016 (7)	0.0025 (7)	0.0018 (8)
C25	0.0381 (9)	0.0531 (11)	0.0410 (10)	−0.0053 (8)	0.0000 (8)	−0.0002 (9)
C26	0.0679 (14)	0.0683 (15)	0.0405 (11)	−0.0041 (11)	0.0055 (10)	−0.0038 (11)
C27	0.0864 (18)	0.0722 (17)	0.0479 (13)	0.0185 (13)	−0.0092 (12)	0.0084 (12)
C28	0.0278 (7)	0.0343 (9)	0.0305 (8)	0.0011 (6)	−0.0035 (6)	−0.0018 (7)
C29	0.0190 (7)	0.0389 (9)	0.0354 (9)	0.0008 (6)	−0.0015 (6)	−0.0028 (7)
O30	0.0273 (5)	0.0451 (7)	0.0439 (7)	0.0007 (5)	−0.0033 (5)	0.0092 (6)

### Geometric parameters (Å, °)

**Table:**

O1—C2	1.3644 (19)	C17—H171	0.962
O1—C7	1.370 (2)	C17—H173	0.972
C2—C3	1.395 (2)	C17—H172	0.999
C2—C29	1.376 (2)	O18—C19	1.429 (2)
C3—C4	1.446 (2)	C19—H191	0.957
C3—C20	1.418 (2)	C19—H193	0.954
C4—O5	1.2540 (19)	C19—H192	0.969
C4—C6	1.464 (2)	C20—O21	1.3594 (17)
C6—C7	1.408 (2)	C20—C22	1.385 (2)
C6—C12	1.424 (2)	O21—H211	0.849
C7—C8	1.384 (2)	C22—C23	1.513 (2)
C8—C9	1.373 (2)	C22—C28	1.409 (2)
C8—H81	0.941	C23—C24	1.503 (3)
C9—O10	1.3550 (19)	C23—H231	0.977
C9—C11	1.413 (2)	C23—H232	0.961
O10—H101	0.837	C24—C25	1.326 (3)
C11—C12	1.387 (2)	C24—H241	0.947
C11—O18	1.377 (2)	C25—C26	1.503 (3)
C12—C13	1.5284 (19)	C25—C27	1.499 (3)
C13—C14	1.503 (2)	C26—H261	0.959
C13—H131	0.950	C26—H263	0.982
C13—H132	0.944	C26—H262	0.969
C14—C15	1.330 (3)	C27—H271	0.952
C14—H141	0.957	C27—H273	0.974
C15—C16	1.503 (3)	C27—H272	0.972
C15—C17	1.509 (3)	C28—C29	1.388 (2)
C16—H161	0.979	C28—O30	1.360 (2)
C16—H163	0.972	C29—H291	0.928
C16—H162	0.968	O30—H301	0.860
			
C2—O1—C7	119.65 (12)	H171—C17—H173	107.0
O1—C2—C3	120.84 (14)	C15—C17—H172	112.2
O1—C2—C29	116.34 (13)	H171—C17—H172	110.8
C3—C2—C29	122.82 (15)	H173—C17—H172	108.1
C2—C3—C4	121.52 (14)	C11—O18—C19	114.47 (14)
C2—C3—C20	116.82 (14)	O18—C19—H191	109.2
C4—C3—C20	121.63 (13)	O18—C19—H193	112.0
C3—C4—O5	119.99 (14)	H191—C19—H193	108.6
C3—C4—C6	116.46 (13)	O18—C19—H192	109.6
O5—C4—C6	123.55 (15)	H191—C19—H192	108.1
C4—C6—C7	117.42 (14)	H193—C19—H192	109.2
C4—C6—C12	125.15 (13)	C3—C20—O21	118.21 (14)
C7—C6—C12	117.41 (14)	C3—C20—C22	122.61 (14)
C6—C7—O1	123.98 (14)	O21—C20—C22	119.18 (14)
C6—C7—C8	122.89 (15)	C20—O21—H211	105.7
O1—C7—C8	113.13 (13)	C20—C22—C23	121.94 (14)
C7—C8—C9	119.11 (14)	C20—C22—C28	117.05 (15)
C7—C8—H81	120.3	C23—C22—C28	121.00 (15)
C9—C8—H81	120.6	C22—C23—C24	112.16 (14)
C8—C9—O10	122.51 (14)	C22—C23—H231	109.8
C8—C9—C11	120.06 (14)	C24—C23—H231	111.0
O10—C9—C11	117.42 (15)	C22—C23—H232	108.3
C9—O10—H101	106.6	C24—C23—H232	108.8
C9—C11—C12	121.07 (15)	H231—C23—H232	106.7
C9—C11—O18	119.40 (14)	C23—C24—C25	127.89 (18)
C12—C11—O18	119.33 (13)	C23—C24—H241	116.0
C6—C12—C11	119.42 (13)	C25—C24—H241	116.1
C6—C12—C13	123.81 (14)	C24—C25—C26	120.8 (2)
C11—C12—C13	116.76 (14)	C24—C25—C27	124.3 (2)
C12—C13—C14	110.75 (13)	C26—C25—C27	114.9 (2)
C12—C13—H131	107.6	C25—C26—H261	110.8
C14—C13—H131	111.4	C25—C26—H263	112.1
C12—C13—H132	109.2	H261—C26—H263	109.3
C14—C13—H132	109.7	C25—C26—H262	109.4
H131—C13—H132	108.1	H261—C26—H262	105.7
C13—C14—C15	127.45 (17)	H263—C26—H262	109.4
C13—C14—H141	114.8	C25—C27—H271	108.1
C15—C14—H141	117.7	C25—C27—H273	111.9
C14—C15—C16	125.16 (19)	H271—C27—H273	110.9
C14—C15—C17	119.7 (2)	C25—C27—H272	108.6
C16—C15—C17	115.16 (19)	H271—C27—H272	107.7
C15—C16—H161	110.2	H273—C27—H272	109.6
C15—C16—H163	111.4	C22—C28—C29	122.40 (15)
H161—C16—H163	109.5	C22—C28—O30	116.51 (15)
C15—C16—H162	109.7	C29—C28—O30	121.09 (14)
H161—C16—H162	106.6	C28—C29—C2	118.29 (13)
H163—C16—H162	109.4	C28—C29—H291	121.4
C15—C17—H171	110.1	C2—C29—H291	120.3
C15—C17—H173	108.5	C28—O30—H301	109.3

### Hydrogen-bond geometry (Å, °)

**Table:**

CgA is the mid-point of the C14═C15 double bond.

**Table:**

*D*—H···*A*	*D*—H	H···*A*	*D*···*A*	*D*—H···*A*
C13—H132···O5	0.94	2.24	2.885 (3)	125
C14—H141···O10^i^	0.96	2.36	3.304 (3)	168
O21—H211···C4	0.85	2.28	2.820 (3)	122
O21—H211···O5	0.85	1.71	2.499 (3)	155
O10—H101···O21^ii^	0.84	1.88	2.691 (3)	164
O30—H301···C14^iii^	0.86	2.56	3.424 (3)	178
O30—H301···C15^iii^	0.86	2.38	3.160 (3)	150
O30—H301···CgA	0.86	2.38	3.227 (3)	166

Symmetry codes: (i) −*x*+3/2, *y*−1/2, *z*; (ii) *x*+1/2, *y*+1/2, −*z*+1/2; (iii) *x*+1/2, *y*−1/2, −*z*+1/2.
